# Face-specific memory deficits and changes in eye scanning patterns among patients with amnestic mild cognitive impairment

**DOI:** 10.1038/s41598-017-14585-5

**Published:** 2017-10-30

**Authors:** Toshikazu Kawagoe, Masateru Matsushita, Mamoru Hashimoto, Manabu Ikeda, Kaoru Sekiyama

**Affiliations:** 1 Graduate School of Social and Cultural Sciences, Kumamoto University, Kumamoto, Japan; 2 Department of Neuropsychiatry, Faculty of Life Sciences, Kumamoto University, Kumamoto, Japan; 3 Faculty of Letters, Kumamoto University, Kumamoto, Japan; 4 Present Address: Department of Neurology, Faculty of Medicine, Shimane University, Shimane, Japan; 5 Present Address: Department of Psychiatry, Osaka University Graduate School of Medicine, Osaka, Japan; 6 Present Address: Graduate School of Advanced Integrated Studies in Human Survivability, Kyoto University, Kyoto, Japan

## Abstract

Amnestic mild cognitive impairment (aMCI) is a prodromal stage of Alzheimer’s disease (AD). Previous studies have shown functional and structural degradation of the fusiform face area, which is a core region for face processing, in addition to medial temporal lobe degradation. We predicted that patients with aMCI exhibit a loss of face processing and/or face memory, accompanied by abnormal eye scanning patterns, since patients who have deficits in face perception (i.e. prosopagnosia) exhibit such tendencies. Eighteen patients with aMCI and age-matched healthy controls were tested for perception and short-term memory of visually presented faces and houses while their gaze was recorded. Patients with aMCI showed a decline in memory, compared with control observers, for faces, but not for houses. Patients looked more at the mouth of faces, compared with control observers. We demonstrate here the loss of short-term face memory in aMCI with abnormal scanning patterns that might reflect the cerebral abnormality found in patients with aMCI.

## Introduction

Individuals with amnestic mild cognitive impairment (aMCI) show cognitive impairment, especially in memory, beyond that expected for their age^1–3^. It is often assumed that aMCI is a transitional state between healthy cognitive aging and dementia, particularly in Alzheimer’s disease (AD). Previous studies suggest that individuals with aMCI progress to AD at a rate of 10–15% per year^3,4^. As there is a high possibility of progression to AD, understanding the characteristics of aMCI is important. In aMCI, the impairment does not affect activities of daily living, so patients tend not to complain about it. However, they show substantial cognitive impairment on evaluation, for example in episodic memory^2^, executive function^5^, and visuospatial ability^6^. Patients at this stage, especially those who are progressing to AD, often go through neurodegenerative changes and/or functional neurological changes^7–10^. There is a substantial loss of grey matter in the medial temporal lobe^9^ of these patients, which may have a negative effect on their memory abilities^11–13^. Whitwell, *et al*.^10^ investigated the progression of cerebral atrophy during the period in between aMCI and AD, by examining the grey matter loss in aMCI three years before the diagnosis of AD. The atrophy was mostly restricted to medial temporal regions including some other anatomical regions such as the amygdala and the fusiform gyrus.

The fusiform gyrus is a part of the occipitotemporal visual extrastriate cortex, which is known for its contribution to face processing^14,15^. This specific region for face processing is called the fusiform face area (FFA). If this area is vulnerable to pathological changes in MCI as Whitwell, *et al*.^10^ suggested, it is reasonable to expect that the face processing system would be impaired in patients with aMCI. Indeed, based on this assumption, Lim, *et al*.^16^ found that patients with aMCI showed deficits in face discrimination using morphed face images in which spatial configuration and colour features had been artificially changed. Patients with aMCI performed slower and less accurately on a simultaneous same-different judgment task than controls. In addition to the degenerative structural change in the fusiform gyrus, a change in functional brain activity also explained the behavioural difference between normal controls and patients with aMCI^5,7,17^. For face recognition tasks, Bokde, *et al*.^18^ reported that the functional connectivity of the FFA to other regions during the face matching task was completely different in aMCI participants compared to age-matched healthy controls, even though task performance and the amount of activation were at the same level. The authors suggested that part of this difference could be due to dysfunction (e.g. decreased connectivity in the visual cortex) and compensation (e.g. increased connectivity in the parietal lobe).

However, the impairment of face recognition in MCI is controversial. Nguyen, *et al*.^11^ attempted to show that patients with MCI had worse memory for unfamiliar faces than healthy controls. They used a set of natural face stimuli to demonstrate such deterioration. They suggested that a face memory test, such as the Wechsler Memory Scale-III (WMS) face test, might differentiate aMCI from normal aging and AD, although their results did not reach the statistical significance. Further, Seelye, *et al*.^19^ investigated the sensitivity of the WMS face test for patients with aMCI and found no significant difference between the scores of normal controls and those of patients with aMCI, even though patients with mild AD had significantly worse scores than the aMCI and healthy control groups.

To the best of our knowledge, studies related to face memory in patients such as those with aMCI investigated either face-name associations or facial expression recognition^13,20,21^. The former would be affected by the general impairment of memory binding in short-term memory observed in patients with AD^12,22^. Although without statistical significance, three studies^11,16,19^ have provided valuable information about face processing and face memory in aMCI. However, impairments in visual short-term memory might also have the potential to be an early diagnostic marker of AD^1^. There are no studies emphasizing the specificity of the impairment with regard to face processing. Moreover, no study ever focused on ‘purer’ short-term memory in aMCI. Nguyen, *et al*.^11^ and Seelye, *et al*.^19^ studied face memory in aMCI, but used a protocol in which participants watch and encode 24 different faces in a single block that tended to cause confusion and resulted in, for example, intrusion errors. Such encoding method might be linked to the distracter and trigger the intrusion, intra-list errors^23^. We aimed to demonstrate here that there is specific degradation of ‘purer’ short-term memory for faces in patients with aMCI using tasks with one-by-one encoding and response. Would the face short-term memory be a sensitive index as a diagnostic marker of aMCI?

In this study, we also used eye-tracking measurements to investigate differences in eye scanning patterns during the tasks. Eye movements are determined by the structure and content of the stimulus as well as by top-down task requirements. Faces are viewed in a typical pattern which is characterized by a focus on the internal facial features, primarily the eyes, but also the nose and the mouth^24–26^, although there are substantial individual differences among healthy adults^27,28^. An association between gaze patterns and face processing has been indicated in face recognition tasks^29,30^ and other visual cognitive tasks^2,31^. While this association is controversial^28,32^, we hypothesized that if patients with aMCI showed a decline in short-term face memory performance, it would be accompanied by a change in eye scanning behaviour. This hypothetical association would be similar to patients with prosopagnosia who show degraded performance in face processing tasks^27,33^. Although the sample sizes were limited due to the participants characteristics, these patients had eye scanning abnormalities characterized by a focus on less informative regions of the face (i.e. the mouth).

To summarize, we intended to demonstrate the behavioural and gaze differences between patients with aMCI and age-matched healthy controls (HC). Based on earlier behavioural, structural, and neurofunctional studies, short-term face memory and other cognitive process could be used as diagnostic markers for aMCI^1,31^. Further, we intended to demonstrate a possible relationship between degraded performance in patients with aMCI and their scanning behaviour of faces.

## Material and Methods

### Participants

A total of 36 participants, 18 in each of two groups, aMCI and HC, were tested in this study. The aMCI participants were recruited at the Memory Clinic of Kumamoto University Hospital (Kumamoto, Japan). The diagnostic criteria laid out by the National Institute for Aging-Alzheimer Association^34^ were applied in this study, which included: (1) Neuropsychological tests and psychological assessments including the Mini-Mental State Examination (MMSE)^35^, the Wechsler Memory Scale-Revised logical memory (WMS-LM), and the Geriatric Depression Scale (GDS)^36^, (2) assessments of activities of daily living, using the Instrumental Activities of Daily Living (IADL) scale and the Physical Self-Maintenance Scale (PSMS)^37^; (3) structural neuroimaging with magnetic resonance imaging (MRI) or computed tomography (CT), functional neuroimaging with single photon emission computed tomography (SPECT); and (4) routine laboratory tests including a complete blood count and metabolic panel, as well as estimation of serum B12, folate, thyroid-stimulating hormone levels, and rapid plasma regain tests. Patients were excluded if they showed evidence of the following: a serious stroke or cortical infarction during either neurological examination or brain imaging, extensive subcortical vascular disease, a space-occupying lesion, or a history of alcohol/substance abuse, major neurological (e.g. traumatic brain injury, normal pressure hydrocephalus) disorders or psychiatric (e.g. schizophrenia, bipolar disorder) illness. The HC group was recruited from elderly people residing in the community through the local club for the aged and through personal connections of its members. None of the participants in the HC group had a diagnosis of neurological or psychiatric illness, underwent current treatment with neuroleptics and orthopaedic medications, or had severe visual and hearing impairments. They were tested on the MMSE, WMS-LM, and GDS. They had MMSE scores above or equal to 25 and WMS-LM scores within 1.5 standard deviations of the Japanese normative data for their age^38,39^. All participants were right-handed except for one in the HC group.

The experiment was undertaken with the understanding and written informed consent of each participant. The study protocol conformed to the Declaration of Helsinki, and was approved by the Kumamoto University Research Ethical Committee.

### Stimuli and task

The face stimuli were neutral faces of young Japanese people (university students), with an equal number of male and female faces. Each face image was derived from a photograph, in full-frontal view, cropped along the face contour, and resized to 560 (height) × 480 (width) pixels. The regions of each eye, nose, and mouth were roughly aligned between images. The images of houses were gathered from the internet and resized to 460 (height) × 365 (width) pixels. Each stimulus was converted to greyscale to prevent judgements based on colour saliency.

We prepared four tasks that tested face perception, house perception, face memory, and house memory. The two perception conditions were performed to assess perceptual abnormalities for faces and objects. In the perception condition, two images (faces or houses) were aligned in a horizontal row and shown simultaneously in the middle of the display after a fixation dot was presented. Participants were asked to respond whether these two stimuli were the same or different by pressing one of two buttons using their left and right index fingers. Two memory tasks were performed for measuring short-term memory for faces and objects. In the memory condition, a study stimulus was presented for 3000 ms following a fixation dot. After the offset, a white blank was presented, with a jitter period between 3000 to 5000 ms. Then a test stimulus pair, similar to the stimuli in the perception task, was presented. Participants were asked to respond whether either (left or right) or neither test stimulus was the same as the study stimulus by pressing one of three buttons. The button for ‘neither’ was pressed with their right thumb. The perception and memory conditions had 14 and 15 trials, respectively. All participants went through the two perception conditions before the two memory conditions. The stimulus category order within each condition was counterbalanced. Each image was presented at most twice for a participant throughout the whole test. The same image did not reappear within the same condition, except in the memory condition (i.e. study stimulus and test stimulus for correct response).

The fixation dot was presented either above or below the stimuli to avoid starting-location biases for face processing^25^. The fixation dot was continuously presented until the participant fixated on it, as confirmed by an experimenter who was monitoring his/her fixation. Participants were orally instructed prior to the task, and were subsequently asked to explain to the experimenter what they needed to do. After confirming that they understood the task, all participants went through practice trials. The stimuli were presented until a response was obtained to avoid putting the participants under pressure. Each experimental task was carried out once the participant went through seven practice trials without making a mistake.

### Eye movement recordings and apparatus

Eye movement data were recorded using the Tobii TX300 (sampling rate was set at 120 Hz; Tobii Technology AB, Sweden) and processed with Tobii Studio (ver. 3.2.1) and MATLAB (R2014a; the Mathworks Inc., Natick, MA). The software was run on a PC with Microsoft Windows 7. Stimuli were presented on a 1920 × 1080 wide screen monitor (96 dpi, 23 in.) equipped with the Tobii eye tracking system. The screen was viewed from a distance of approximately 57 cm under free viewing conditions; the face images were presented at a visual angle of just under 15° × 12°, the distance between eye and mouth was approximately 7° in height and house images were displayed in 12° × 9° rectangle shapes. A nine-point-calibration was performed prior to the test to calculate the exact eye position for each participant.

The Tobii Studio software can visualize eye movement data with the gaze superimposed on the stimulus and export raw eye movement data in numerical values that can be processed with MATLAB. To define fixations, we adapted the Tobii Studio I-VT filter. This filter classifies eye movements based on the velocity of the directional shift of the eye. Parameters relating to this filter were set by default and provide accurate fixation classification for common eye tracking data collections. In short, the velocity calculator estimated the eyes’ angular velocity for each data point by dividing the angular difference between the preceding and subsequent data point by the time interval between them. The calculator window length was set to 20 ms by default. Then, the I-VT fixation classifier, which had threshold functions driven by arguments from the velocity calculator, classified data points with an angular velocity below the threshold value (30 degrees/second as a default) as “fixation” and data points above as “saccade”. For more details, with references, about this filter, please refer to the Tobii Studio user manual (https://www.tobiipro.com/siteassets/tobii-pro/user-manuals/tobii-pro-studio-user-manual.pdf).

### Analyses

Behavioural performance was measured as the proportion of correct responses (PC) and reaction time (RT). Firstly, to verify that the task was performed appropriately, we ran a binomial test to exclude participants who had performance levels equal to chance (thresholds: 0.64 for two-alternative forced-choice and 0.5 for three-alternative forced-choice). However, none of our participants was excluded by applying this criterion. Some of the participants with aMCI asked to know which button corresponded to the answer, during an ongoing trial. We performed a Smirnov-Grubbs test on RTs, to exclude such extended trials from all analyses. The test excluded only 2.3% and 2.0% (aMCI and the HC group), and 2.6% and 1.9% (aMCI and the HC group) of the trials from the perception conditions and memory conditions, respectively.

Participants who asked about the relationship between the buttons and the answer more than twice during the whole experiment, or who did not understand the relationship at all, were excluded from the RT analyses. Three participants with aMCI were excluded by these criteria, although they were encouraged to answer orally during the session. Following this procedure, the data from 15 participants with aMCI and 18 with HC was entered into the RT analyses.

Eye movement data were calculated for three Areas of Interest (AOI) that were defined by rectangles of equal size corresponding to the eye, nose, and mouth regions. Two indices for eye movement data were recorded: fixation duration and the number of fixations. Fixation duration represented the total time of fixation for each AOI. It measured the sum of the duration (ms) for all fixations within each AOI for all test stimuli throughout the experiment. The fixation duration was summed for the two faces in the perception condition. The number of fixations, literally, represented the sum of the number of fixations in each AOI for all test stimuli throughout the experiment. We analysed the proportion of fixation counts for each AOI (number of fixations in each AOI/total number of fixations). The duration for each fixation was calculated as the fixation duration, divided by the number of fixations. This measure provided an index that would not be affected by the RT of the participants. Participants whose eye movements could not be captured were excluded from the eye data analyses, while their RT and accuracy data were used. Two and five participants were excluded in the HC and aMCI group, respectively. The excessive lack of eye movement data was presumably due to the use of corrective lenses such as lined bi- or tri-focal glasses, to excessive blinking during the test, or to frequent dropping of eyelids due to biological aging. For all statistical tests, the significance level α was set at 0.05. For post-hoc multiple comparisons after ANOVA, Ryan’s method was used.

## Results

The characteristics of the participants are shown in Table 1. There were no significant differences between the two groups in the scores as tested by Student’s t-tests (*p* < 0.01), except for the MMSE, WMS-LM I, and WMS-LM II.

**Table 1:** Characteristics of the participants.

	aMCI participants	HC participants
N (N of men)	18 (8)	18 (5)
Age	77.61 (5.32)	74.05 (16.66)
Education	12.44 (2.33)	13.00 (1.97)
MMSE	24.22 (3.90) *	28.11 (1.64)
WMS-LM I	2.50 (2.03) *	9.22 (3.70)
WMS-LM II	1.00 (1.88) *	7.66 (4.02)
GDS	3.56 (2.09)	2.35 (2.02)

Note: Data (except for N) are shown as mean (standard deviation). Asterisks on aMCI data indicate significant differences as compared with HC data (*p < 0.01). aMCI, amnestic mild cognitive impairment; HC, healthy controls, N, number of individuals; MMSE, mini-mental state examination; WMS-LM, Wechsler memory scale logical memory test (I: immediate recall, II: delayed recall); GDS, Geriatric Depression Scale.

### Behavioural data

**Figure 1:**
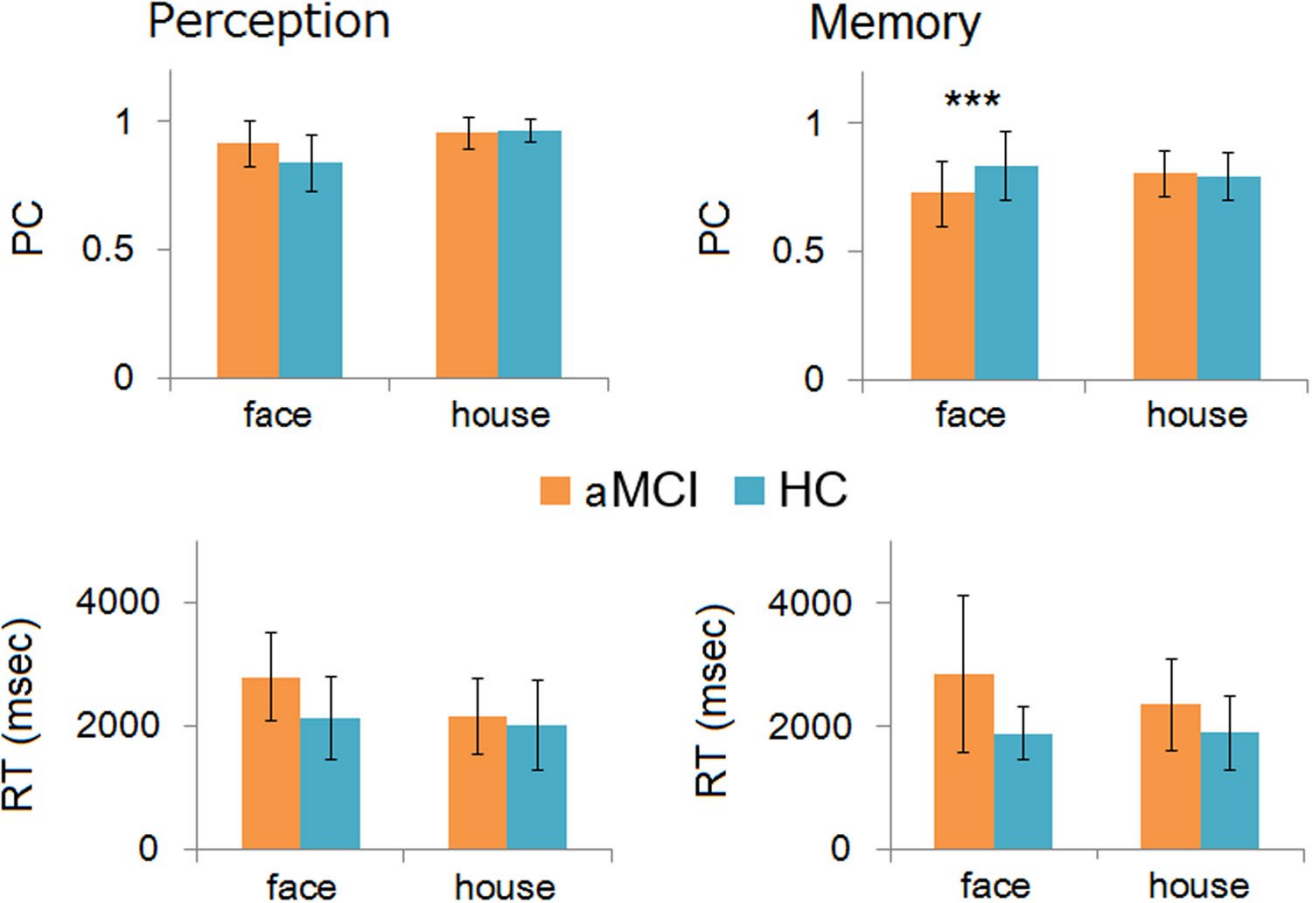
Behavioural performance (reaction time and proportion of correct responses) for each group, shown as the mean across participants. Note that the three-way interaction (group × condition × stimulus) was significant for the proportion of correct responses (PC; upper row) and the two-way interaction (group × stimulus) was significant for reaction time (RT; lower row). Error bars show standard deviation and asterisks indicate significant simple effect in three-way interaction (***p < 0.001).

For the behavioural data, we analysed PC and RT for each condition, shown in Fig. 1. We conducted a three-way ANOVA (group: aMCI or HC, condition: perception or memory, stimuli: face or house), because we aimed to clarify the specific deterioration of face-memory performance in patients with aMCI, which could be demonstrated through a three-way interaction.

#### Proportion of correct responses

We observed a significant three-way interaction between group, condition and stimulus (*F*(1,34) = 9.46, *p* < 0.001, *η* ^2^_*p*_ = 0.22). Post-hoc tests indicated that the simple interaction of group × condition for face stimuli was significant (*F*(1,68) = 15.82, *p* < 0.001, *η* ^2^_*p*_ = 0.19). This was mediated by the significant simple effect of condition in the aMCI group for face stimuli (*F*(1,68) = 34.09, *p* < 0.001, *η* ^2^_*p*_ = 0.33) indicating a lower PC for face memory than for face perception. On the contrary, the simple effect of condition in the HC group for face stimuli was not significant (*F*(1,68) = 0.05, *p* = 0.831, *η* ^2^_*p*_ < 0.001). These results for accuracy clearly show the specificity of face memory impairments in patients with aMCI. In addition, the three-way interaction remained significant when participants who were excluded following eye movement analyses were removed (*F*(1,24 = 4.68, *p* = 0.041, *η* ^2^_*p*_ = 0.16).

#### Reaction time

The same three-way ANOVA tests were conducted for RT data. This analysis showed that the three-way interaction was not significant (*F*(1,31) < 0.01, *p* = 0.995), however, a two-way interaction between group and stimulus was significant (*F*(1,31) = 5.26, *p* = 0.028, *η* ^2^_*p*_ = 0.15). This significant interaction was mediated by the delayed response for face stimuli in the aMCI (*F*(1,62) = 12.70, *p* < 0.001, *η* ^2^_*p*_ = 0.17), compared with the HC group. These performance patterns were not caused by a speed-accuracy trade-off because there was no positive correlation between RT and PC for each data set. The analysis of RT indicated deterioration of face processing in the aMCI group. Eliminating the participants who were excluded following the eye movement analyses did change the results. Neither the interaction nor the main effect was statistically significant (*p*s > 0.05).

### General eye movement data

**Figure 2:**
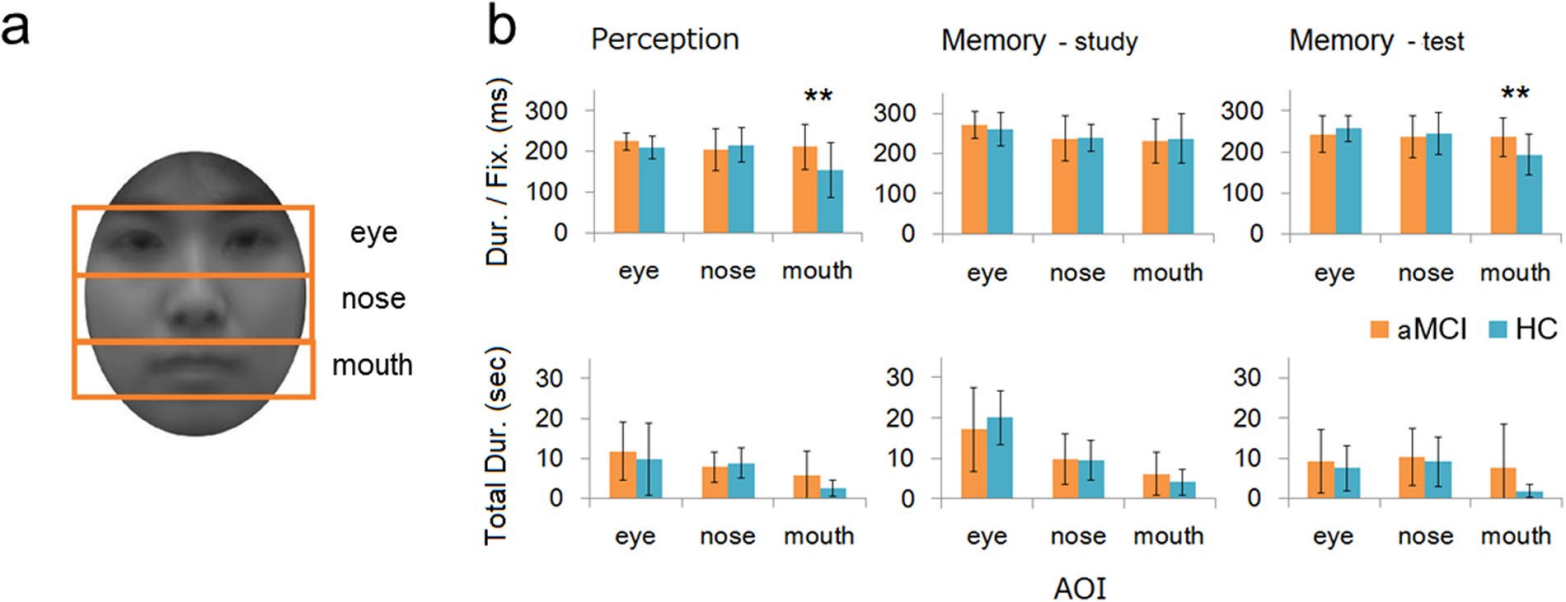
(**a**) Illustration of each area of interest (AOI) on an averaged face image. (**b**) Eye fixation duration for each AOI. Fixation duration per fixation (upper row) and Total fixation duration (lower row) are visualized separately for each condition. Error bars show standard deviation and asterisks indicate the simple main effect of group in two-way interaction (**p < 0.005).

We did not focus on house stimuli for eye movement data analyses, because the spatial components were not aligned between stimuli. For analyses of eye tracking data for faces, three indices (number of fixations, total fixation duration, and duration per fixation) were considered for each AOI (eyes, nose and mouth; Fig. 2a). First, we showed the duration per fixation because this index is independent from participants’ RT. Then, we show the number of fixations for each AOI, which was calculated as a proportion of the total fixation across all AOIs, and total fixation duration, which would be affected by RT.

#### Duration per fixation

We calculated the duration per fixation (Fig. 2b, upper row). A two-way (group × AOI) ANOVA for each condition showed a significant interaction between group and AOI in the perception (*F*(2,50) = 4.849, *p* = 0.011, *η* ^2^_*p*_ = 0.162) and memory-test (*F*(2,46) = 3.810, *p* = 0.029, *η* ^2^_*p*_ = 0.142), but not in the memory-study condition. These interactions were mediated by longer fixations to the eyes and nose relative to the mouth for perception and memory-test conditions in only the HC group (The significant simple main effects of AOI in the HC group were as follows: for perception, *F*(2,50) = 7.132, *p* = 0.002, *η* ^2^_*p*_ = 0.221; for memory-test, *F*(2,46) = 9.016, *p* < 0.001, *η* ^2^_*p*_ = 0.281). The significant AOI differences in the HC group were as follows: for perception, eyes > mouth, *p* = 0.012; nose > mouth, *p* < 0.001; for memory-test, eyes > mouth, *p* < 0.001; nose > mouth, *p* = 0.001). In the memory-study condition, a significant main effect of AOI was confirmed (*F*(2,46) = 4.075, *p* = 0.023, *η* ^2^_*p*_ = 0.150); both groups made longer fixations to the eyes as compared to the nose (*p* = 0.026) and the mouth (*p* = 0.011).

#### Number of fixations

The number of fixations data for each AOI as percent to the whole screen was analysed (Table 2). For each condition, we tested independence using chi-squared tests. Only in the memory-test condition was a significant relationship between group and AOI found (χ^2^ = 6.17, *p* = 0.022, *Cramer’s V* = 0.18). Residual analyses for each condition indicated a fixation shift toward the mouth in the aMCI compared with the HC group.

**Table 2:** Proportion of number of fixations for each area of interest in total number of fixations.

*Proportion of fixation* (*%*)	Perception			Memory – study			Memory – test		
	*eye*	*nose*	*mouth*	*eye*	*nose*	*mouth*	*eye*	*nose*	*mouth*
MCI	45.8	32.4	21.6	50.5	30.9	18.5	35.6	39.3	24.9*
HC	46.2	40.1	13.7	56.3	31.3	12.3	39.2	49.2	11.4

Note: Asterisks indicate the data which was significantly greater than the counterpart by residual analyses (*p < 0.01). MCI, mild cognitive impairment; HC, healthy controls.

#### Total Fixation duration

Fixation duration was analysed (Fig. 2b, lower row). Two-way (group × AOI) ANOVAs were conducted for each condition separately because we assumed fixation behaviour between these conditions to be qualitatively different. These analyses did not reveal any significant interactions in any condition. A main effect of group was confirmed only in the memory-test condition (*F*(1,27) = 9.15, *p* = 0.005, *η* ^2^_*p*_ = 0.25). However, this was caused by the longer RT in MCI participants, verified by a correlation analysis (*r* = 0.485, *p* = 0.012). Analyses revealed a significant main effect of AOI in every condition (perception: *F*(2,54) = 7.80, *p* = 0.001, *η* ^2^_*p*_ = 0.22; memory-study: *F*(2,54) = 20.03, *p* < 0.001, *η* ^2^_*p*_ = 0.43; memory-test: *F*(2,54) = 3.46, *p* = 0.038, *η* ^2^_*p*_ = 0.11). Post-hoc Ryan’s tests revealed that the fixation duration in each AOI was significantly different (perception: longer fixation on eyes (*p* < 0.001) and nose (*p* = 0.016) than mouth; memory-study: longer fixation on eyes and nose (*ps* < 0.001) than mouth and on nose than mouth (*p* = 0.021); memory-test: longer fixation on nose than mouth (*p* = 0.011)).

### Initial eye movement data

In addition to these results, we conducted a time-course analysis for eye movement data for the memory condition. A previous study suggested that optimal face encoding is achieved with the initial two fixations during the study phase, and that performance does not improve with additional fixations^26^. Based on these findings, we aimed to demonstrate the difference in initial ‘important’ fixations between the groups during the study phase. In line with Hsiao and Cortell’s report^26^ and due to the nature of the tasks (i.e., a comparison between simultaneously displayed stimuli), we did not consider differences in initial fixations in the perception condition and the test phase in the memory condition.

**Figure 3:**
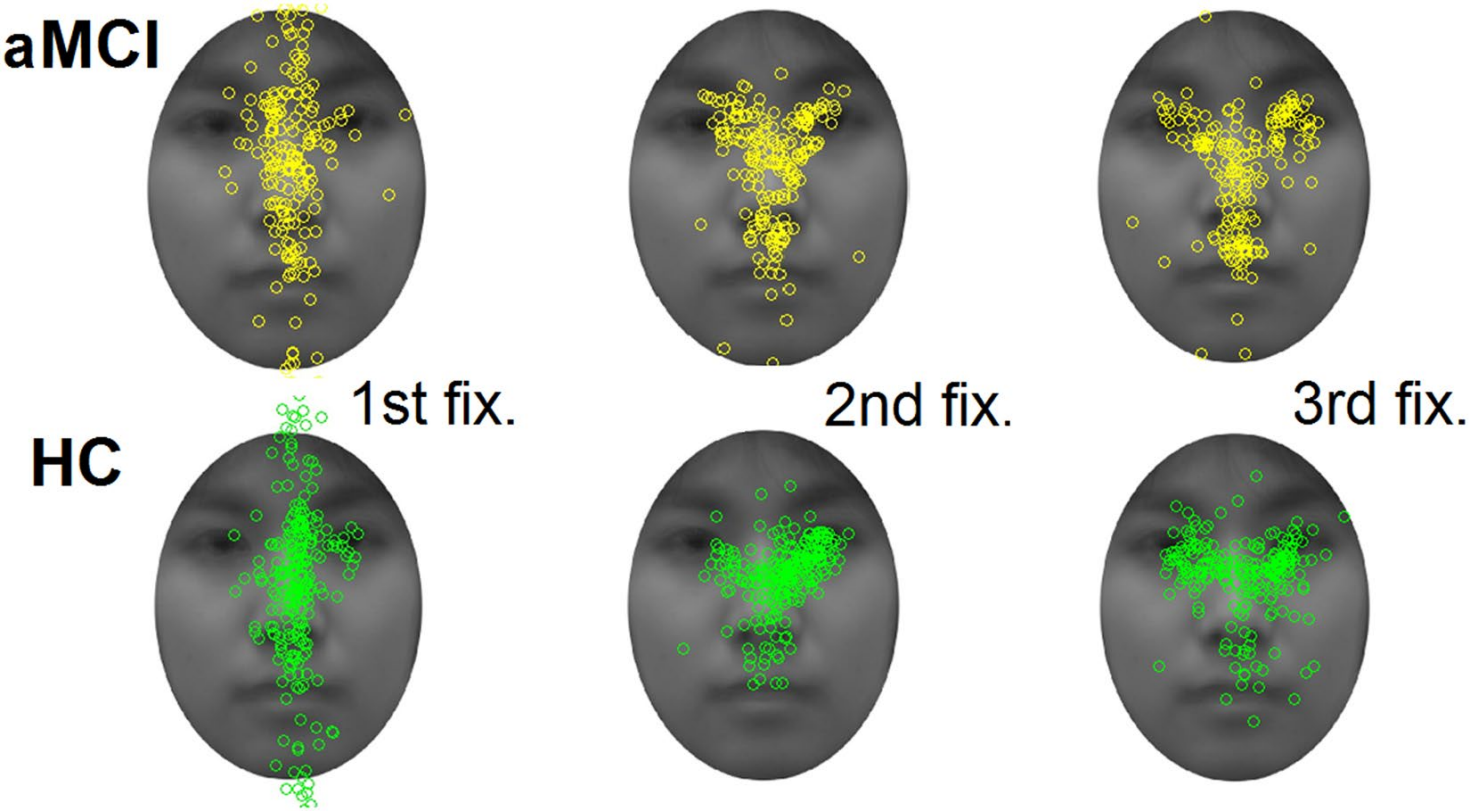
Landing points of the three initial fixations during the study phase in the memory condition were superimposed on an averaged face image. In each of the first (left panels), second (middle panels), and third fixation (right panels) plots, a single dot represents one fixation by one participant in one trial.

Since our participants were older adults, we extended the range from the first to the third fixation, taking slower general reaction speed into account. Therefore, the first, second, and third fixations from the stimulus onset of the study phase in the memory condition were analysed for each AOI (Fig. 3). Fixation duration was calculated and analysed using a two-way ANOVA (group: aMCI or HC; order: first, second, third). The analysis only showed a main effect of order (*F*(2,54) = 20.41, *p* < 0.001, *η* ^2^_*p*_ = 0.435), which was brought about by a shorter first fixation compared to the second (*p* < 0.001) and third (*p* < 0.001), replicating the results of Hsiao and Cottrell^26^. The landing location depicted in Table 3 and Fig. 3, was analysed with a chi-square test in a 2 (group) × 3 (AOI) design. There was no statistical difference between the groups for the first and second fixation for each AOI (*ps* > 0.05). However, for the third fixation, landing points were significantly differently between the groups (χ^2^ = 10.38, *p* = 0.002, *Cramer’s V* = 0.23); the aMCI group fixated more often on the mouth (*p* < 0.001) and less often on the eyes (*p* < 0.001) than the HC group.

**Table 3:** Proportion of the number of initial three fixations for each area of interest during the study phase of the memory condition.

*Proportion of fixation* (*%*)	1st fix			2nd fix			3rd fix		
	*eye*	*nose*	*mouth*	*eye*	*nose*	*mouth*	*eye*	*nose*	*mouth*
MCI	50.3	36.4	13.2	57.1	31.5	11.4	55.1	25.4	19.4**
HC	49.2	41.1	9.7	62.8	32.9	4.2	71.4*	22.9	5.6

Note: Asterisks indicate the data which is significantly larger than the counterparts by residual analyses (*p < 0.05, **p < 0.01). MCI, mild cognitive impairment; HC, healthy controls.

## Discussion

The present study aimed to demonstrate face-specific short-term memory impairments in participants with aMCI. As we expected, behavioural results showed face-specific impairments in the aMCI group especially for performance indexed by PC. A three-way interaction showed that the participants in the aMCI group had lower PC for face as compared to house stimuli, and in the memory as compared to the perception condition. RT data was partly in accordance with that of PC and indicated the aMCI group’s impairment in processing face stimuli. The face-specific deficit became more evident in PC when memory load was added to the task. The removal of subjects excluded due to excessive eye movement did not appear to affect this result. The hypothesis of this present study was based on negative structural changes of the lateral fusiform area including the FFA^10^, and its altered functional connections to other important areas for face processing^18^ in patients with aMCI. Further, Jonas, *et al*.^40^ recently demonstrated the causal role of the anterior fusiform gyrus in face recognition via brain stimulation techniques. Also, we used natural human faces, instead of morphed face images^16^, and our stimuli have thus real inter-individual differences. The observed group differences for natural faces could strengthen the hypothesis regarding face-related ability loss in patients with aMCI.

An important result of this study relates to the differences between the groups with respect to the numbers of fixations in each AOI. The aMCI group showed a greater number of fixations in the region of the mouth compared to the HC group in the memory-test condition. This was also true for the memory-study condition, as in the results of the time-course analyses. When memorizing a face, people focus their gaze onto the region of the eyes or that between the eyes and the nose^26,32^. Studies have shown that this is the best way to produce an optimal performance, as this is the most informative area of the face^29,30^, supported by the finding that the eyes, but not the mouth, can be a clue for face recognition^41^. Hsiao and Cottrell^26^ have demonstrated in young participants that this eye-attention pattern emerges from the second fixation onward. We replicated their results in healthy older adults at the third fixation, but found a different pattern in the aMCI group, in which this did not emerge. A clue as to why the aMCI group showed such a fixation pattern can be obtained from reports about a patient with acquired prosopagnosia, who exhibited a stronger fixation bias towards the mouth^33^. This patient, with a pure form of prosopagnosia, relied on the lower part of the face, particularly information from the mouth, in the encoding phase of face recognition tasks, presumably due to an impairment in holistic processing. Participants with aMCI might avoid fixating on the eye region, which is important for holistic processing^42^, due to impaired holistic processing abilities caused by structural and/or functional changes in the focal region (i.e. FFA). Future work may examine this prediction using inverted faces to determine if the group differences obtained in the current study are due to abnormalities in holistic processing^43^. Another possibility is that our participants were not able to use the strategy to focus on the most informative region when they memorized the face stimuli presented. Although we did not precisely define the cause of the shift of fixations to the mouth area, the difference between the groups indicates that participants with aMCI scanned face images in a less optimal way than those in the HC group.

The index of total fixation duration was not closely coupled with the difference in performance, maybe because of the large variability in duration. This index might not be sensitive enough to detect the difference in scanning behaviour between the two groups. However, duration per fixation analyses indicated a shorter duration per fixation on the mouth in the HC relative to the aMCI group, during the perception condition and in the test phase in the memory condition. This result suggests that fixation on the mouth is not so important when comparing the stimulus presented with one in memory in HC.

The present study verified that participants with aMCI have impaired face memory and altered eye scanning patterns for faces. These findings give some direction to clinical applications, for example regarding the usefulness of short-term face memory tasks as a screening tool. A prior study that used the face test in WMS III failed to show a statistically significant difference between participants with MCI and HC^11,19^. However, our study supports the possibility that short-term face memory tasks allow discrimination between patients with aMCI and healthy older adults. To increase the explanatory power of the present study, we might have to assess a ‘purer’ kind of short-term face memory. In clinical situations, monitoring the patients’ eye behaviour during a task might aid the assessment. To the best of our knowledge, this is the first study to elucidate face-specific memory impairments in older adults with aMCI by using natural faces and an eye tracking technique. Lastly, we do recognize that there are some limitations in this study. We used the same images for the “same” and “old” judgement in the current tests. This may be an issue, especially in the perceptual condition, because it may enable participants to use an image matching, rather than facial recognition strategy. However, the the three-way interaction on PC might be independent to this issue. Also, we did not investigate the association between behaviour and the structural or functional state of patients’ brains in this study. Future studies might avoid this shortcoming and reveal the association between deficits in face memory and scanning patterns.
